# Mental health and resilience quotient of patients in primary care units during the widespread COVID-19 pandemic in Thailand: a cross-sectional study

**DOI:** 10.1186/s13030-023-00298-x

**Published:** 2024-02-27

**Authors:** Tanyalak Sanphiboon, Napakkawat Buathong, Rattanaporn Chootong

**Affiliations:** Department of Family and Preventive Medicine, Faculty of Medicine, Prince of Songkla University, 15 Karnjanavanich Road, Hat Yai, Songkhla, 90110 Thailand

**Keywords:** COVID-19 pandemic, Mental health, Primary care unit, Resilience

## Abstract

**Background:**

The coronavirus disease 2019 pandemic impacted both the physical and mental health of individuals. The resilience quotient (RQ) is an important factor that decreases mental health problems. This study aimed to explore mental health problems and RQ in patients who visit Primary Care Units (PCU).

**Methods:**

A cross-sectional study was conducted on participants aged 18 to 60 years who visited the PCU of Songklanagarind Hospital from May 1, 2022, to June 31, 2022. Participants completed a self-administered questionnaire on baseline characteristics and the Primary Care Assessment, Personal Resource Questionnaire, satisfaction with life scale, Thai RQ, PHQ-9, and GAD-7, and the results were analyzed by descriptive, logistic regression, and Spearman’s rank correlation.

**Results:**

Among the 216 participants, 72.2% were female, and the median age was 39 (24,51) years old. Most of them had normal RQ levels (61.1%). Of these, 4.2% and 12.1% exhibited moderate to high levels of depression and anxiety, respectively. This study found that sex (OOR 1.93; 95% CI 1.01–3.74), age (OOR 1.03; 95% CI 1.01–1.06), moderate and high social support levels (OOR 9.51; 95% CI 3.36–28.85), and a high life satisfaction level (OOR 4.67; 95%CI 1.75–13.25) were associated with RQ. Moreover, the results showed that ≥ 3 times visiting PCU (β 1.73; 95% CI 0.39–3.08), BMI (β 0.13; 95% CI 0.04–0.23) and experiencing stressful events (β 2.34; 95% CI 1.32–3.36) were positively associated with depression. Finally, experiencing stressful events (OR 4.1; 95% CI 1.09–15.47) significantly affected anxiety, however, moderate and high life satisfaction levels acted as a protective factor against anxiety (OR 0.19; 95% CI 0.07–0.54 and OR 0.03; 95% CI 0.01–0.16, respectively).

**Conclusion:**

Although there were a few patients with moderate to severe levels of depression and anxiety, most of them had normal RQ levels. However, there were some patients with low RQ levels which correlated to a high risk of psychiatric diseases such as depression and anxiety. Healthcare providers should focus on interventions that enhance resilience in both proactive and defensive strategies to reduce negative mental problems during these formidable times.

## Background

At the end of 2019, the world faced the coronavirus disease 2019 (COVID-19) pandemic. Thailand had many cases of infection and a death toll exceeding 33,000 [1]. Due to this pandemic, the government announced “locked down” and “social distancing” policies that affected many dimensions, such as the economic and healthcare systems, increasing the burden and complexity of management of physicians, and delayed treatment of other diseases [2]. Additionally, it also affected mental health, particularly depression and anxiety. In 2020, during the COVID-19 pandemic, the prevalence of anxiety increased from 6.33% to 50.9% and that of depression increased from 14.6% to 48.3% in the general population and healthcare providers [3–5]. However, there is limited research focusing on non-communicable diseases (NCDs), patients who were affected by the difficulty of using care services, neglected treatment, changes in lifestyle behavior patterns, etc. [6].

Resilience quotient (RQ) refers to the emotional and mental potential of an individual to learn and adapt when confronted with difficult situations. Individuals who have this ability can bounce back from a negative experience to normal functioning and overcome critical crises which may lead to mental illness [7]. RQ can affect the mental health of the general population positively [8] and can help NCD patients control the disease [9, 10]. Varying results of RQ have been found in different population groups, such as a cancer group that had lower RQ when compared to a group without cancer [11]. Most of the NCD patients in one study had normal to high RQ levels [12], however, some studies found that NCD patients had low RQ levels [13].

During the pandemic, few studies were conducted on mental health problems, RQ, or associated factors in patients in primary care settings. Consequently, this study aimed to explore these factors in the patients visiting the Primary Care Unit (PCU) of Songklanagarind Hospital and compared the results of patients with or without NCDs.

## Methods

### Study design and setting

This cross-sectional study was conducted on 216 participants aged 18 to 60 years who visited the PCU, Songklanagarind Hospital between May 1, 2022, and June 31, 2022. Its aim is to explore the prevelance of mental health problems, RQ, and associated factors in this patient sample.

### Participants

To participate in this study, the participants had to meet the following inclusion criteria: patients aged 18 to 60 years, able to read and understand Thai language. The exclusion criteria were as follows: patients who had emergency conditions that required abrupt treatment, pregnant women, and patients who were previously diagnosed with psychological disorders.

**Fig. 1:**
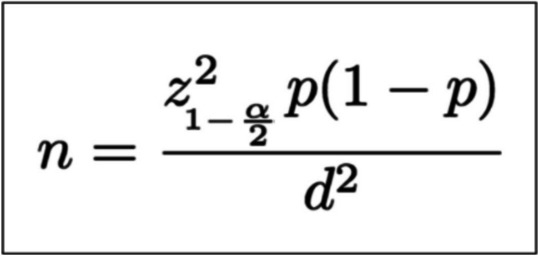
Infinite population proportion formula

The participants were sampled by the infinite population proportion, as presented in Fig. 1, by setting proportion (p) = 0.5, error (d) = 0.07, and alpha (α) = 0.05, which could be calculated for 196 participants.

### Questionnaire for assessment

The self-administered questionnaire contained six sections. The first section collected demographic characteristics: age, sex, marital status, education level, occupation, income, adequacy of income, religion, underlying diseases, duration of underlying disease, BMI, smoking and alcohol drinking status, number of times visiting the PCU, postponement of PCU, and stressful events.

The second section collected data required to assess trust in physicians through the Primary Care Assessment (PCAS), which included eight items. The total score ranged between 8 and 40 points and was divided into three groups: low (score 8–18), moderate (score 19–28), and high (score ≥ 29). The Cronbach’s alpha coefficient was 0.72.

The third section collected data needed to assess social support through the Personal Resource Questionnaire (PRQ). The PRQ by Brand and Weinert [14], translated into Thai language by Sarinrat Tangchurat, was utilized to measure the social support level. The total score ranged between 0 and 100 points and was divided into three groups: low, moderate, and high social support levels. The Cronbach’s alpha coefficient was 0.85 [15].

The fourth section collected data needed to assess life satisfaction through the Satisfaction with Life Scale (SWLS), by Diener, Emmons, Larsen, and Griffin [16], translated into Thai language by Isara Boonyarit [17]. The total score ranged between 5 and 35 points and can be divided into seven groups based on the score, however, the researcher regrouped these into three groups: low (5–19 points), moderate (20–25 points), and high (26–35 points). The Cronbach’s alpha coefficient was 0.78.

The fifth section assessed the patient’s resilience. The questionnaire was developed by the Department of mental health [18] and had a Cronbach’s alpha coefficient of 0.75. Patient resilience was divided into three groups: low (< 55 points), normal (55–69 points), and high (> 69 points).

The sixth section evaluated mental health (depression and anxiety). The PHQ-9, developed by Lotrakul Meetrakool [19], was used to measure depression levels and included nine items. The Cronbach’s alpha was 0.79. Depression levels were divided into three groups: low (0–14 points), moderate (15–19 points), and high (20–27 points). The Generalized Anxiety Disorder-7 (GAD-7) was used to measure the anxiety level,with the scores divided into three groups: low (0–9 points), moderate (10–14 points), and high (15–21 points) [20]. The sensitivity and specificity were 0.89 and 0.82, respectively.

### Data collection

After obtaining permission through informed consent, the researcher allowed the participants to take the self-administered questionnaire, which took approximately 30 min per individual to complete. If the participants did not want to answer any questions, they could withdraw from the study at any time. Anonymity was ensured through the questionnaire not requiring the participants to include their names or hospital numbers, and the data was saved and coded in Microsoft Excel 2019 to prevent persons not involved in this study from accessing the data.

### Analyses

Demographic data, psychological domain, RQ level, depression level, and anxiety level were described, distributed, and analyzed by using a Mann–Whitney U test for continuous data and a Chi-squared test or Fisher’s exact test for discrete data. The categorical data are shown as percentages. Regarding continuous data, if there was a normal distribution, the mean and standard deviation (SD) are presented. If there was abnormal distribution, the median and interquartile range (IQR) are presented.

Regression analysis was used to analyze the associated factors. All variables which had a *p*-value of less than 0.2 were included in the univariate analysis. Backward stepwise elimination was used to create the final models. The RQ-associated factors were analyzed using ordinal logistic regression indicated the ordinal odd ratio (OOR). The depression-associated factors were analyzed using linear regression and anxiety was analyzed using binary logistic regression. Collinearity was examined using the Variance inflation factor (VIF). Correlations between RQ and depression and anxiety were analyzed using Spearman’s rank correlation.

## Results

A total of 216 participants were included in this study. Table 1 presents the baseline characteristics of the participants. Most were female (72.2%), single (50.9%), Buddhist (84.3%), and had graduated with a bachelor’s degree (51.4%). The median age was 39 (24–51) years old, and the median BMI was 24.1 (21.0, 27.9) kg/m^2^. The top three occupations were university student (25.9%), government official (14.4%), and businessman (14.4%). The median income was 465.63 (289.6, 1,054.9) USD/month, and 74.5% reported adequacy of income. Most reported that they had never smoked, nor had they ever drunk alcohol. Of the 216 participants, 131 (60.6%) were NCD patients, most of whom had dyslipidemia, hypertension, or diabetes mellitus. The participants indicated that they had visited the PCU clinic over the past 6 months once (45.8%) or twice (35.6%). Most did not postpone doctor’s appointments. Additionally, 49.5% indicated that they had had a stressful event, most of which concerned the COVID-19 pandemic.

**Table 1:** Baseline characteristics

Variables	Patients (*n* = 216)
**Sex, n (%)**	
Female	156 (72.2)
Male	60 (27.8)
**Age (years), Median (Q1, Q3)**	39 (24,51)
**Status, n (%)**	
Single	110 (50.9)
Married	91 (42.1)
Divorced	15 (7.0)
**Education level, n (%)**	
Less than primary school	13 (6.0)
Highschool/Vocational certificate	40 (18.5)
Diploma/High vocational	27 (12.5)
Bachelor’s degree	111 (51.4)
Greater than bachelor’s degree	25 (11.6)
**Income (USD/month), Median (Q1, Q3)**	465.6 (289.6, 1,054.9)
**Income adequacy, n (%)**	
Yes	161 (74.5)
**BMI (kg/m**^**2**^**), Median (Q1, Q3)**	24.1 (21.0, 27.9)
**Occupation, n (%)**	
University student	56 (25.9)
Government official	31 (14.4)
Seller/Businessman	31 (14.4)
Maid	16 (7.4)
Officer	11 (5.0)
Agriculturalist or fisherman	9 (4.2)
State enterprise employee	6 (2.8)
Others	56 (25.9)
**Religion, n (%)**	
Buddhism	182 (84.3)
Islam	26 (12.0)
Christianity	5 (2.3)
No religion	3 (1.4)
**Non-communicable diseases (NCDs), n (%)**	
Yes (multiple responses)	131 (60.6)
Dyslipidemia	71 (54.2)
Hypertension	45 (34.4)
Diabetes Mellitus	28 (21.4)
Chronic kindey disease	2 (1.5)
Stroke	2 (1.5)
COPD or Asthma	2 (1.5)
**Smoking, n (%)**	
Current	12 (5.5)
Former smoker	14 (6.5)
Never	190 (88.0)
**Alcohol consumption, n (%)**	
Current	42 (19.4)
Former drinker	14 (6.5)
Never	160 (74.1)
**Visiting PCU clinic (previous 6 months), n (%)**	
0 times	4 (1.9)
1 time	99 (45.8)
2 times	77 (35.6)
≥ 3 times	36 (16.7)
**Postponement of doctor’s appointment (previous 6 months), n (%)**	
Yes	30 (13.9)
**Stressful event, n (%)**	
Yes	107 (49.5)
Concerning the COVID-19 pandemic	49 (22.7)
Relationship problems	20 (9.3)
Unemployment problems	12 (5.6)
Loss of a lover	9 (4.2)
Litigation problems	4 (1.9)
Other	39 (18.1)

*COPD* Chronic obstructive pulmonary disease, *BMI* Body mass index, *PCU* Primary Care Unit, *n* sample, *NCDs* Non-communicable diseases, *Q1* Quartile 1, *Q3* Quartile 3, *kg* kilogram, *m *meter

### Psychological domain and mental health

Table 2 presents the results for the psychological domain and mental health. It was observed that most of the participants were in the high-level group (85.2%) regarding trust in physicians, in the moderate-level group (69.4%) regarding social support, and there was no difference between patients with NCDs and those without NCDs. Regarding life satisfaction, 112 (51.8%) of the participants indicated a high level of life satisfaction, which significantly differed in proportion (*p* < 0.001) between the with and without NCDs groups. Regarding RQ, depression, and anxiety, the median RQ score was 66.0 (61.0–70.0) and most of the participants indicated a normal RQ level (61.1%). A significant difference was observed in the proportion of low, normal, and high levels between participants with and without NCDs (*p*-value 0.037). The median score of depression was 5 (2–9), 95.2% of the participants indicated a low level of depression, and there was no difference between the groups. The median score for anxiety was 3 (0–6), with most of the participants indicating a low level of anxiety (87.9%).

**Table 2:** Psychological domain, resilience quotient, and mental health status

Variables	Total (*n* = 216)	With NCDs (*n* = 131)	Without NCDs (*n* = 85)	*p*-value
**Trust in physician, n (%)**				0.345^a^
Low	-	-	-	
Moderate	32 (14.8)	17 (13.0)	15 (17.6)	
High	184 (85.2)	114 (87.0)	70 (82.4)	
**Social support, n (%)**				0.055^a^
Low	30 (13.9)	22 (16.8)	8 (9.4)	
Moderate	150 (69.4)	83 (63.4)	67 (78.8)	
High	36 (16.7)	26 (19.8)	10 (11.8)	
**Life satisfaction, n (%)**				< 0.001^a^
Low	38 (17.6)	17 (13.0)	21 (24.7)	
Moderate	66 (30.6)	31 (23.7)	35 (41.2)	
High	112 (51.8)	83 (63.3)	29 (34.1)	
**Resilience quotient, n (%)**				0.037^a^
Median (Q1, Q3) = 66 (61, 70)				
Low	17 (7.9)	7 (5.4)	10 (11.8)	
Normal	132 (61.1)	76 (58)	56 (65.8)	
High	67 (31.0)	48 (36.6)	19 (22.4)	
**Depression, n (%)**				0.752^c^
Median (Q1, Q3) = 5 (2, 9)				
Low	207 (95.8)	126 (96.2)	81 (95.2)	
Moderate	6 (2.8)	4 (3.0)	2 (2.4)	
High	3 (1.4)	1 (0.8)	2 (2.4)	
**Anxiety, n (%)**				0.009^c^
Median (Q1, Q3) = 3 (0, 6)				
Low	190 (87.9)	121 (92.4)	69 (81.2)	
Moderate	22 (10.2)	7 (5.3)	15 (17.6)	
High	4 (1.9)	3 (2.3)	1 (1.2)	

a = Chi-squared test, b = Mann–Whitney U test, c = Fisher’s exact test, NCDs = non-communicable diseases, n = sample, Q1 = Quartile 1, Q3 = Quartile 3

### Associated factors of RQ level

Table 3 presents factors associated with the RQ level. The variables significantly associated with the RQ level were sex, age, social support, and life satisfaction. Male participants had a higher level of RQ than did female participants (OOR 1.93, 95% CI 1.01–3.74). Increased age had a higher RQ level (OOR 1.03, 95% CI 1.01–1.06). Regarding social support, moderate to high levels of social support were associated with a higher RQ level (OOR 9.51, 95% CI 3.36–28.85). Regarding life satisfaction, a high level of life satisfaction was associated with a higher RQ level (OOR 4.67, 95% CI 1.75–13.25). Marital status, educational level, and life satisfaction (moderate level) were not significantly associated with RQ.

**Table 3:** Ordinal logistic regression of factors associated with the RQ level

Variables	Crude OOR (95% CI)	Adjusted OOR (95% CI)	*p*-value
**Male** (Ref: female)	1.53 (0.84, 2.78)	1.93 (1.01, 3.74)	0.04*
**Age** (years)	1.03 (1.02, 1.06)	1.03 (1.01, 1.06)	0.02*
**Marital status** (Ref: single or divorced)			
Marital status	1.44 (0.84, 2.48)	0.63 (0.31, 1.27)	0.20
**Education level** (Ref: less than bachelor’s degree)			
Bachelor’s degree or greater	0.74 (0.42, 1.28)	0.85 (0.44, 1.62)	0.61
**Social support** (Ref: low level)			
Moderate and high level	11.38 (4.45, 31.04)	9.51 (3.36, 28.85)	< 0.001*
**Life satisfaction** (Ref: low level)			
Moderate level	2.42 (0.97, 6.25)	1.30 (0.50, 3.47)	0.60
High level	11.32 (4.60, 29.60)	4.67 (1.75, 13.25)	0.002*

*OOR* Ordinal odds ratio, *95% CI* 95% confidence interval, *Ref.* reference

* = *p*-value was significant (< 0.05)

### Factors associated with depression score and anxiety level

Table 4 presents the factors associated with the depression score. They include the number of visits to the PCU ≥ 3 times (β 1.73, *p*-value 0.012), having any stressful events (β 2.34, *p*-value < 0.001), BMI (β 0.13, *p*-value 0.007), having an NCD (β -2.21, *p*-value < 0.001), moderate to high social support level (β -2.07, *p*-value 0.01), and moderate and high life satisfaction level (β -1.66, *p*-value 0.035 and β -3.59, *p*-value < 0.001, respectively).

**Table 4:** Linear regression of factors associated with the depression score

Variables	Crude coefficient (95% CI)	Adjust coefficient (95% CI)	*p*-value
**Visits to the PCU clinic (previous 6 months)**			
(Ref: 0–2 times)			
≥ 3 times	2.59 (0.97, 4.22)	1.73 (0.39, 3.08)	0.012*
**Stressful event** (Ref: No stressful events)			
Having any event	3.38 (2.22, 4.53)	2.34 (1.32, 3.36)	< 0.001*
**BMI**	0.05 (-0.06, 0.17)	0.13 (0.04, 0.23)	0.007*
**Underlying diseases** (Ref: No NCDs)			
Having an NCD	-2.85 (-4.06, -1.64)	-2.21 (-3.29, -1.13)	< 0.001*
**Social support** (Ref: low level)			
Moderate and high levels	-3.54 (-5.26, -1.81)	-2.07 (-3.65, -0.49)	0.01*
**Life satisfaction** (Ref: low level)			
Moderate level	-2.87 (-4.48, -1.25)	-1.66 (-3.21, -0.11)	0.035*
High level	-5.89 (-7.33, -4.4)	-3.59 (-5.17, -2.02)	< 0.001*

*95% CI* 95% confidence interval, *Ref.* Reference, *PCU* Primary Care Unit, *NCDs* Non-communicable diseases, *BMI* Body mass index

* = *p*-value was significant (< 0.05)

Factors associated with the anxiety level are presented in Table 5 and were analyzed using binary logistic regression. They include having had a stressful event (OR 4.1, *p*-value 0.037) and having a moderate or high level of life satisfaction, which was protective against a high anxiety level (OR 0.19, *p*-value 0.002 and OR 0.03, *p*-value < 0.001, respectively). The duration of an underlying disease was not associated with the anxiety level.

**Table 5:** Binary logistic regression of factors associated with a higher anxiety level

Variables	Crude OR (95% CI)	Adjusted OR (95% CI)	*p*-value
**Stressful event (Ref** **: ** **No stressful events)**			
Having any event	6.79 (2.25, 20.47)	4.1 (1.09, 15.47)	0.037*
**Duration of underlying disease**	0.88 (0.77,1)	0.90 (0.79, 1.03)	0.125
**Life satisfaction (Ref** **: ** **low level)**			
Moderate level	0.19 (0.07, 0.51)	0.19 (0.07, 0.54)	0.002*
High level	0.03 (0.01, 0.12)	0.03 (0.01, 0.16)	< 0.001*

*OR* Odds ratio, *95% CI* 95% confidence interval, *Ref.* referenc

* = *p*-value was significant (< 0.05)

### Correlations among RQ, depression, and anxiety

The correlations among RQ, depression, and anxiety are presented in Table 6. The associations were tested using Spearman’s rank correlation. The RQ was significant and moderately negatively correlated with depression and anxiety (ρ -0.53 and -0.57, respectively). Depression was significant and moderately positively correlated with anxiety (ρ 0.7).

**Table 6:** Correlation between mental health status and resilience quotient

Variables	Median	Q1, Q3	Resilience	Depression	Anxiety
Resilience	66	61, 70	-	-	-
Depression	5.0	2, 9	- 0.53 (< 0.001)*	-	-
Anxiety	3.0	0, 6	- 0.57 (< 0.001)*	0.70 (< 0.001)*	-

Bivariate correlation analysis was performed using Spearman’s rank correlation**:** ρ (*p*-value)

Q1 = Quartile 1, Q3 = Quartile 3, * = *p*-value was significant (< 0.05)

## Discussion

This study was conducted during the second wave of the COVID-19 pandemic in Thailand and reports low prevalences of both depression and anxiety status. Of the participants, 4.2% had a moderate to high level of depression (2.8% moderate and 1.4% high) and 12.1% had a moderate to high level anxiety level, which was lower than the prevalence in a previous study that showed high prevalences of depression (22.13% moderate and 17.38% high) and anxiety (22.9% moderate and 13.6% high) in the Thai population and lower than other Southeast Asian countries such as Singapore, Malaysia, and Indonesia [21]. These results may be explained by the fact that most participants in this study had moderate to high levels of life satisfaction and social support (82.4% and 86.1%, respectively), which is associated with decreases in depression and anxiety scores. Moreover, it can be explained by the successful attempt of the hospital in developing holistic clinical practice guidelines for the care of our patients. Most of the participants in this study had a normal or high RQ level, which is inconsistent with a previous study in Thailand during 2020 that found 43.9% of the participants had a low resilience quotient level, with a mean score on the Brief Resilient Coping Score (BRCS) of 13.9 (SD 4–20) [22]. This difference could be from the timing of the collection the data that suggested the RQ was changeable and adapted along with time.

This is the first research in done Southeast Asia that compares the mental illness and resilience of patients with or without an NCD. In this study, there was no differences in the depression scores of participants with or without an NCD, although a previous study found an increased prevalence of depression during the lockdown in both groups, but not reported the significance [23]. Although having an NCD would be expected to increase the risk of mental illness, surprisingly, this study found that having an NCD was negatively associated with the depression score. There were significant differences in the proportions of mild, moderate, and high levels of anxiety between participants with and without an NCD. The proportion with a moderate anxiety level was higher in the without NCD group, which may be explained by the fact that most of the participants in this group were university students who adapted to online learning. This may have created a more anxious and restricted environment, similar to that of the previous study, which evaluated 1,000 tertiary students during the COVID-19 pandemic and found that 20.4% had a moderate level and 6.6% a high level of anxiety [24]. These anxiety levels had an emotional impact, learning impact, financial impact, social impact, and technological impact [25]. The proportion of low, normal, and high RQ levels differed in both the with and without NCD patients. Most of the NCD patients had a high RQ level, which was consistent with a study reporting that the more lethal the disease, the higher the patient’s resilience to reduce the negative effect [10]. Furthermore, the results may be explained by the fact that NCD patients in this PCU may have a good doctor-patient relationship, as most of the NCD patients indicated a high level of trust in their physicians. Thus, the findings are consistent with previous study findings indicating that doctor-patient relationships following the precepts of patient-centered care are a significant resource that can lead to increased patient resilience [26].

This study found that sex, age, social support, and life satisfaction were RQ-associated factors. Male participants had a higher RQ level than female participants, which is consistent with previous studies [13, 27]. A higher age indicated a higher RQ level, which is consistent with a previous study of resilience after the experience of trauma that found that older age was associated with a higher resilience level. This may be explained by older adults having more experience and having developed better coping abilities, which assist them in adverse life-changing situations [28]. Participants with moderate to high social support levels had a higher RQ level, which is consistent with previous studies [29–31]. Moreover, these results may be explained by the fact that most of the participants indicated a high level of trust in their physician, which strongly correlated with informational and emotional support (*r* = 0.542) [32]. A high life satisfaction level was positively associated with the RQ level, which is consistent with previous studies [30, 33].

In this study’s linear regression of factors associated with depression, variables positively associated with depression scores included the number of visits to the PCU, BMI, and having had a stressful event. The number of visits to the PCU ≥ 3 times over the past 6 months was positively associated with depression, which may be explained by the fact that the increasing number of visits to the PCU may indicate that patients had active physical illnesses or diseases. This finding is consistent with previous studies, which found a strong correlation between disease activity and depression in patients with rheumatoid arthritis [34] and in a study that found that elevation in symptoms of depression was associated with increased active inflammatory bowel disease (OR 2.7; 95% CI 1.15–6.34) [35]. In this study, an increase in BMI indicated a higher depression level score, which is consistent with previous studies that found that obesity increased the risk of depression [36, 37]. Having any stressful event was positively associated with depression, which is consistent with a previous study that found that different stressors, such as socioeconomic-related stressors, impacted psychological problems [38]. Interestingly the most often reported stressful event was concerning in the COVID-19 pandemic but the result of mental illness, such as depression and anxiety, was quite lower than expected. This could be from our medical service being in a tertiary setting, which may have raised confidence in our care.

Variables that were negatively associated with depression scores included NCDs, social support, and life satisfaction. Having any NCD was negatively associated with the depression score, which may be explained by the fact that most of this study’s NCD participants indicated moderate and high levels of trust in their physician, indicating the importance of the impact of the therapeutic relationship. This finding was consistent with a previous study, which suggested that trust in primary care physicians among patients with diabetes mellitus (DM) was associated with lower levels of depression and anxiety [39]. Moreover, 94.6% of the participants in the NCDs group had a normal or high RQ level, indicating that most of the NCD participants had optimal protection against depression. Moderate and high social support levels were negatively associated with depression, which is consistent with a previous study that found that less social support can increase the risk of depression [40]. Moderate and high life satisfaction levels were negatively associated with the depression score, which is consistent with a previous study that found a moderate negative correlation between life satisfaction and depression (β -0.41, *p*-value 0.001) [41]. Factors associated with anxiety levels included stressful events and life satisfaction. Having experienced any stressful event resulted in a higher anxiety level, which is consistent with a previous study that found that different stressors such as socioeconomic-related stressors had an impact on psychological problems [38]. Both moderate and high life satisfaction levels were found to be protective against a higher anxiety level, which is consistent with a previous study that found a negative association between anxiety and life satisfaction (β -0.26, *p*-value 0.01) [42].

RQ was moderately correlated with both depression and anxiety (ρ 0.53 and 0.57 respectively), which is consistent with previous studies [28]. COVID-19 infection was a worldwide emerging disease, which may be related to the results of studies of mental illness and RQ showing little difference. Results from Thailand may differ because it is a developing country that has a wide range of socioeconomic status. Our research was conducted in a tertiary hospital and most of the patients are doing well socioeconomically, thus the results may not be able to be generalized to the whole Thai population.

### Strengths of the study

This research was conducted during the second wave of the COVID-19 outbreak, which allowed for the collection of information concerning patient RQ and mental health status. Furthermore, this study also examined factors associated with RQ, depression, and anxiety, which can assist healthcare teams to acknowledge the problem and allow them to be vigilant for the occurrence in patients with and without NCDs. There were few studies conducted on RQ during the COVID-19 pandemic in Thailand, thus, this study’s results would be helpful for healthcare workers caring for patients.

### Limitation

This study collected data through the use of self-administered questionnaires that included 90 items. The large number of items may have caused participants to feel exhausted and could, consequently, have led to mistakes in filling out the information, which may have caused information bias. In addition, data on this population was not collected before the COVID-19 pandemic, making it impossible to compare the results between the two periods.

### Suggestion

Many other factors can predispose a patient to anxiety and depression that could not be assessed due to the wide variability of factors examined in this study. They include vaccination status and knowledge about COVID-19 infection, factors that should be further studied in the future.

## Conclusions

Amidst the COVID-19 pandemic, few participants exhibited a moderate to severe level of depression and anxiety and most participants had a normal resilience quotient level. However, some patients had a low resilience quotient, which correlated to a high risk of developing a psychiatric disease such as depression or anxiety. Consequently, the enhancement of resilience should be prioritized as a preventative measure against mental illness through early detection and both proactive and defensive strategies. Our hospital has systems and teams that are taking such action. If we find a patient at risk, they are quickly given counselling to enhancing resilience and they are continuously followed to reduce guard against negative mental problems during these formidable times.

## Data Availability

The datasets used and analyzed during the study are available from the corresponding author upon reasonable request.

## Abbreviations

BRCS: Brief resilient coping score
CAD: Coronary artery disease
CD-RISC 10: Connor-davidson resilience scale 10
CD-RISC 25: Connor-davidson resilience scale 25
COPD: Chronic obstructive pulmonary disease
CKD: Chronic kidney disease
DLP: Dyslipidemia
DM: Diabetes mellitus
NCDs: Non-communicable diseases
PCU: Primary care unit
RQ: Resilience quotient
VIF: Variance inflation factor
WHO: World health organization
